# Primary hepatic Epstein–Barr virus-positive diffuse large B-cell lymphoma associated with azathioprine immunosuppression: a case report

**DOI:** 10.1186/s13256-023-03907-z

**Published:** 2023-05-02

**Authors:** Paulina S. Marell, Min Shi, Majken T. Wingo

**Affiliations:** 1 Department of Medicine, Division of Community Internal Medicine, Geriatrics, and Palliative Care, Mayo Clinic, Rochester, MN USA; 2 Department of Laboratory Medicine and Pathology, Division of Hematopathology, Mayo Clinic, Rochester, MN USA

**Keywords:** Lymphoma, Immunosuppression, Azathioprine, Case report

## Abstract

**Background:**

Hepatic masses are relatively common findings, and the diagnostic approach often begins by identifying patient and mass characteristics that are risk factors for malignancy. Chronic immunosuppression is a known risk factor for various malignancies, and azathioprine in particular has been reported in association with solid and hematologic malignancies, including diffuse large B-cell lymphoma.

**Case presentation:**

A 46-year-old white woman presented to clinic with several weeks of gastrointestinal symptoms and was found to have a hepatic mass on imaging. Her history was notable for neuromyelitis optica spectrum disorder on chronic immunosuppression with azathioprine. It was initially thought to be an inflammatory adenoma. On 6-month follow-up imaging, the mass had grown rapidly in size and was surgically resected. Further workup determined the mass to be an iatrogenic immunodeficiency-associated Epstein–Barr virus-positive diffuse large B-cell lymphoma confined to the liver. Azathioprine was discontinued and the patient underwent treatment with rituximab with no evidence of recurrence 2 years after the initiation of treatment.

**Conclusions:**

This case report describes the first time hepatic Epstein–Barr virus-positive diffuse large B-cell lymphoma has been reported with azathioprine, which highlights the unique sequelae of chronic immunosuppression, including atypical hematologic malignancies, and the importance of considering chronic immunosuppression in the diagnostic evaluation of a hepatic mass.

## Background

Ultrasound performed for right upper quadrant abdominal pain may uncover an incidental or causal hepatic mass [1]. Evaluating a hepatic mass requires obtaining a history and performing a physical examination, and may involve imaging tests and a review of pathology obtained through biopsy [2]. The diagnostic approach typically begins with an assessment for the presence of risk factors for hepatocellular carcinoma or hepatic adenoma, a history of malignancy, elevated tumor markers, or weight loss. If those factors are absent and a cystic lesion is found on ultrasound, further imaging recommendations are based on characteristics of the mass. If a solid mass is found, guidelines recommend obtaining further imaging with computed tomography (CT) or magnetic resonance imaging (MRI) [2].

Chronic immunosuppression has been identified as a risk factor for various malignancies [3–5] and impacts the pretest probability of malignancy when a new hepatic mass is identified. Specifically, diffuse large B-cell lymphoma, a subtype of non-Hodgkin’s lymphoma, is associated with exposure to immunosuppressive drugs as well as inherited immunodeficiency disorders [6–9]. Azathioprine is an immunosuppressive agent in the antimetabolite category that affects purine nucleotide synthesis [10]. It can be used for rejection prophylaxis in kidney and other transplants and to treat a variety of autoimmune conditions, such as autoimmune hepatitis, systemic lupus erythematous, and rheumatoid arthritis [10]. It has been associated with both solid and hematologic malignancies, with previous research suggesting a dose–response relationship between cumulative azathioprine dose and risk of malignancy [11]. Among the hematologic malignancies, previous reports have suggested an association between azathioprine and acute myeloid leukemia [12], myelodysplastic syndrome [13, 14], and lymphoma, including non-Hodgkin’s lymphoma [15–18]. A cohort study among people with inflammatory bowel disease found that use of thiopurines, which include azathioprine, resulted in a multivariate-adjusted hazard ratio of greater than 5 for development of lymphoproliferative disorders [19]. Case reports have been published of diffuse large B-cell lymphoma (DLBCL) developing in the setting of azathioprine use within the gastrointestinal tract [20, 21], the genitourinary organs [22, 23], the central nervous system [24], and the skin [25].

## Case presentation

A 46-year-old white woman presented to the internal medicine clinic with several weeks of worsening symptoms of esophageal reflux, a pressure-like sensation in the abdomen, early satiety, intermittent nausea, and bloating. Her medical comorbidities at that time included seronegative neuromyelitis optica spectrum disorder with bilateral optic neuritis resulting in legal blindness and transverse myelitis resulting in neuropathic pain and neurogenic bladder/bowel treated with low-dose prednisone and azathioprine for the past 12 years. She also had Henoch–Schönlein purpura in childhood transiently requiring dialysis, chronic kidney disease, gastro–esophageal reflux disease, obstructive sleep apnea, pernicious anemia, subclinical hypothyroidism, hypertension, asthma, and herpes simplex labialis. Her surgical history included a laparoscopic cholecystectomy 8 years prior. Her family history is limited to lung cancer in a paternal grandfather. Her medications included amlodipine, azathioprine, duloxetine, fluticasone–salmeterol, gabapentin, hydrochlorothiazide, hydroxyzine, norethindrone, omeprazole, oxybutynin, prednisone, simvastatin, and valacyclovir. Physical examination was notable for moderate abdominal tenderness most prominent in the epigastrium and right upper quadrant.

**Fig. 1:**
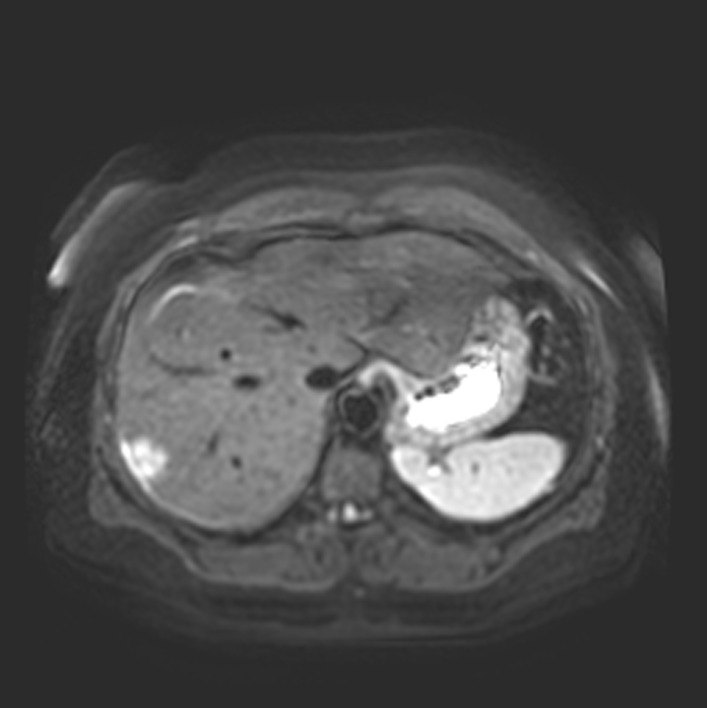
Initial abdominal MRI. In the periphery of the right hepatic lobe there is a somewhat ill-defined lobulated region of T2 hyperintensity about 2.3 cm in size. Centrally, the signal is similar to the hepatic parenchyma. This area has heterogeneous restricted diffusion

A right upper quadrant ultrasound was obtained, which showed increased hepatic echogenicity suggestive of hepatic steatosis and a 1.9 × 2.4 × 1.9 cm hypoechoic solid mass in the right lobe of the liver. An MRI was pursued to further characterize the hepatic mass (Fig. 1). This showed an ill-defined lobulated region of T2 hyperintensity corresponding to the mass visualized on ultrasound. Gastroenterology performed a chart review consultation, and they felt that imaging was compatible with an inflammatory adenoma. The patient was recommended to lose 20–25 pounds over the next 6 months and repeat abdominal imaging at that time.

**Fig. 2:**
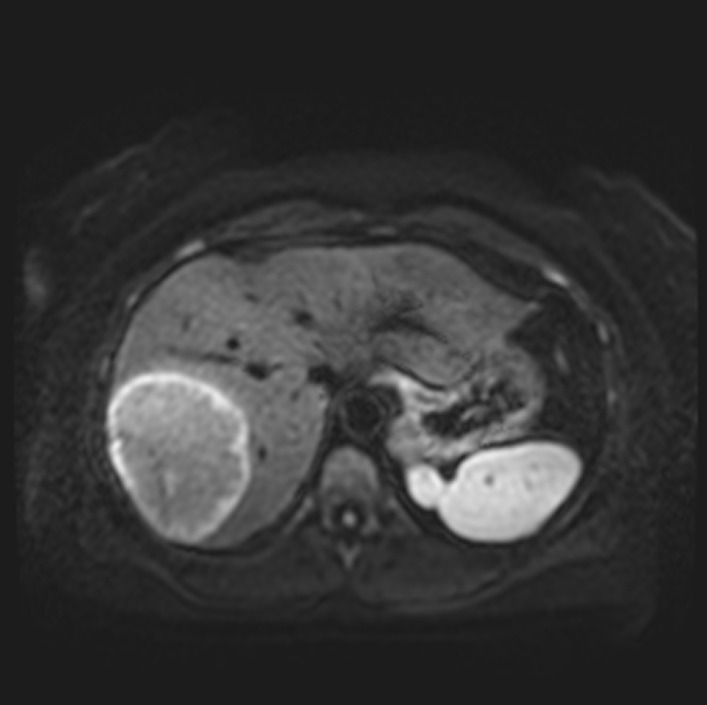
Subsequent abdominal MRI at 6-month follow-up. There is a 9.3 × 7.0 × 9.4 cm circumscribed mass in the right lobe of the liver. It increased substantially in size since 11/07/2019, when it measured 2.3 cm. The mass does not contain lipid. There is a small central cystic or necrotic area. No biliary ductal dilatation and no overlying capsular retraction

**Fig. 3:**
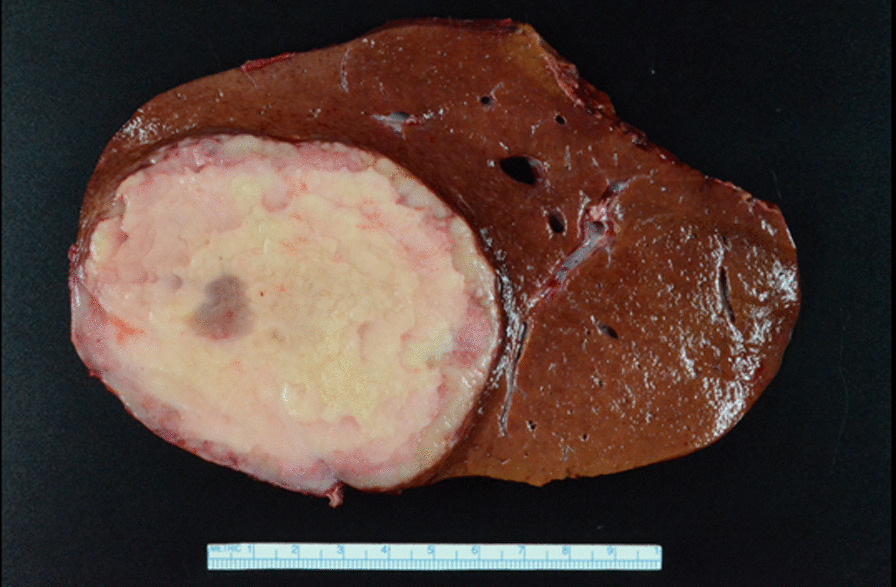
Surgical specimen. A single 9.5 × 9.2 × 8.2 cm well-circumscribed, tan–white mass with abundant central necrosis is present 4.7 cm from the surgical margin. The mass involved the right lateral aspect of the liver

**Fig. 4:**
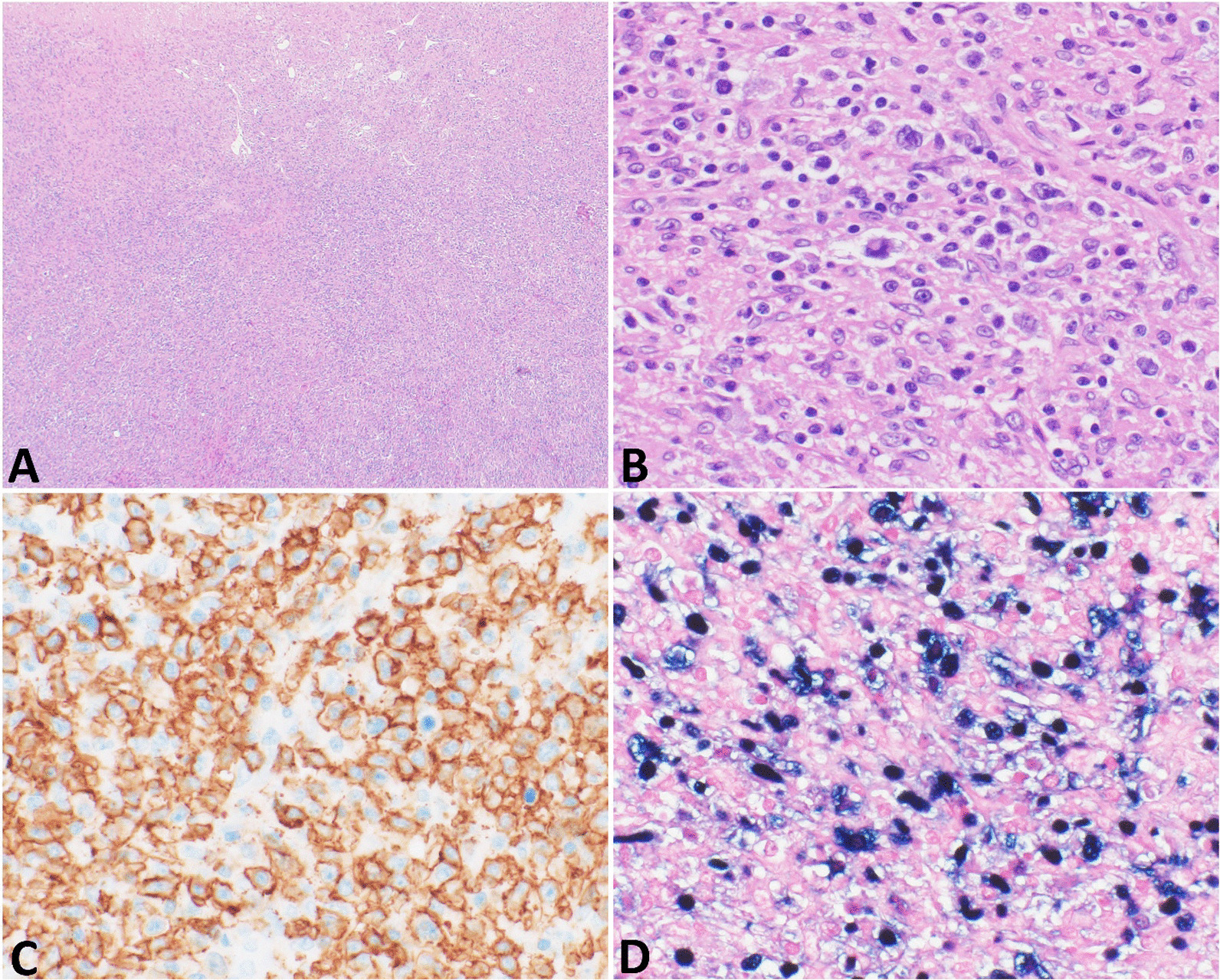
Histologic specimen. Pathological features of the liver mass. **A** Histologic sections show liver parenchyma is diffusely infiltrated by an atypical lymphoid population with necrosis. Magnification ×40. **B** The atypical lymphoid cells have large-sized nuclei, irregular nuclear contours, vesicular chromatin, distinct nucleoli, and moderate amounts of cytoplasm. Background reactive small lymphocytes and histiocytes are present. Magnification ×400. **C** The large, atypical lymphocytes show immunoreaction with CD20. **D** They are diffusely positive for EBV. Magnification ×400

**Fig. 5:**
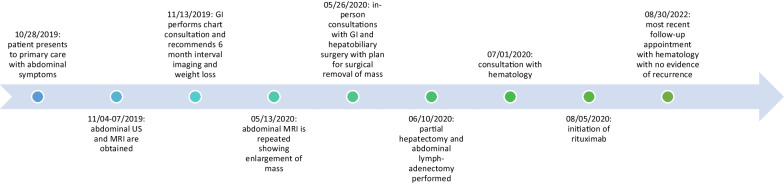
Timeline of patient course. Timeline of patient course from October 2019 to August 2022

Six months later, a repeat abdominal MRI was pursued. This was notable for a rapid increase in the size of the mass now measuring 9.3 × 7.0 × 9.4 cm with a small central cystic or necrotic area (Fig. 2). No abdominal lymphadenopathy was noted. Approximately 1 month later she underwent a right partial hepatectomy and abdominal cavity lymphadenectomy (Fig. 3). Pathological evaluation of the hepatic mass found infiltration of the liver parenchyma with a large abnormal lymphoid population with multiple areas of extensive necrosis. Further histologic testing indicated an Epstein–Barr virus (EBV)-positive, CD20-positive DLBCL with no *MYC* gene rearrangement and a non-germinal center B-cell phenotype (Fig. 4). Evaluation of the resected lymph nodes was negative for malignancy. A bone marrow biopsy was performed, which was negative for lymphomatous involvement of the bone marrow. A positron emission tomography–computed tomography (PET–CT) scan was performed, which showed no abnormal fluorodeoxyglucose (FDG)-uptake to suggest an extra-hepatic site involved by lymphoma. Treatment was initiated with discontinuation of azathioprine and initiation of rituximab infusions (1 g each) with two doses initially 2 weeks apart and two additional doses 6 months later to complete four total doses. After completion of rituximab, she transitioned to surveillance. On her most recent abdominal imaging 2 years after the initiation of treatment, she had no evidence of recurrent disease. A timeline of the patient’s course is presented (Fig. 5).

## Discussion

To our knowledge, azathioprine-associated primary hepatic EBV-positive DLBCL has not previously been described in the literature, though DLBCL in other sites has been described in the setting of azathioprine use, as previously discussed. This patient was initially thought to have a hepatic adenoma due to multiple risk factors, which highlights the importance of maintaining a broad differential diagnosis when evaluating a patient with a new hepatic mass. Categorization of possible diagnoses comprises cystic lesions, including simple hepatic cysts, polycystic liver disease, hydatid cysts (echinococcosis), biliary cystadenoma, and hepatic abscesses; benign solid masses, including hepatic adenomas, focal nodular hyperplasia, hemangiomas, and angiolipomas; malignant solid masses, including hepatocellular carcinoma, cholangiocarcinoma, primary lymphoma, angiosarcoma, hepatoblastoma, and hepatic metastases; and focal fatty liver, which, while not a true mass, may present as such on imaging [2, 26, 27]. This patient had risk factors for multiple diagnoses, such as female sex, oral contraceptive use, and obesity increasing her risk for a hepatic adenoma [28] and chronic immunosuppression increasing her risk for a malignant lesion. Other risk factors for a malignant lesion include older age, cirrhosis, hepatitis B and C infections, primary sclerosing cholangitis, obesity, tobacco use, and alcohol use [29, 30]. Indications for a biopsy of the liver lesion include confirming a diagnosis of nodular regenerative hyperplasia, inconclusive imaging in suspected hepatic adenoma, and if a malignant lesion is suspected and biopsy would change management [2]. Importantly, hepatocellular carcinoma can be diagnosed with greater than 90% accuracy with imaging alone when a lesion is at least 2 cm in size [31].

This case also demonstrates the importance of serial imaging, not only to detect interval growth of a lesion, as was found in this case, but also to detect changes in imaging characteristics. The initial MRI image obtained of the lesion in this case appeared compatible with a focal area of infection or inflammation. Follow-up MRI 6 months later was interpreted to most likely represent a hepatic adenoma with possible transformation to hepatocellular carcinoma, ultimately found to be lymphoma. Primary hepatic lymphoma is rare, representing less than 1% of all extra-nodal lymphomas [32]. It has been found to mimic cholangiocarcinoma [33] and acute fulminant hepatitis [34]; as such, it can be difficult to diagnose.

In brief, DLBCL is a B-cell lymphoma that is one of the most common lymphoid malignancies in adults and can arise de novo or as transformation of a less aggressive lymphoma [6, 7, 35]. It should be considered in patients with a rapidly enlarging, asymptomatic mass at a nodal or extranodal site. Initial management depends on age, stage of disease, subtype, and comorbidities. Typical first-line therapy includes rituximab, cyclophosphamide, doxorubicin, vincristine, and prednisone (R-CHOP) with or without radiation. Five-year survival for all ages is about 63%. DLBCL could arise from the setting of immunodeficiency/immune dysregulation. Potential causes for immunosuppression include congenital immunodeficiencies, acquired immunodeficiency, or iatrogenic immunodeficiency. Most cases are associated with EBV. Reduction of the level of immunosuppression is likely to be an important part of any therapy regimen [36]. In this case, per the 5th edition of the World Health Organization Classification of Haematolymphoid Tumors: Lymphoid Neoplasms, this could be classified as a DLBCL, EBV-positive, associated with immune deficiency/dysregulation [37].

## Conclusion

This case illustrates the unique sequelae that can occur from chronic immunosuppression, in this case from an iatrogenic source. Although rare cases of hematologic malignancies have been seen in chronic azathioprine use, a diagnosis of primary hepatic EBV-positive DLBCL in an immune deficiency/dysregulation setting is also possible. This report bolsters the recommendation for clinicians caring for patients on chronic immunosuppression to consider atypical malignancies when evaluating a new symptom or finding. In summary, this report describes an index case of primary hepatic EBV-positive DLBCL associated with chronic immunosuppressive therapy with azathioprine in a patient with neuromyelitis optica spectrum disorder.

## Data Availability

Not applicable to this manuscript.

## Abbreviations

DLBCL: Diffuse large B-cell lymphoma
CT: Computed tomography
MRI: Magnetic resonance imaging
EBV: Epstein–Barr virus
PET–CT: Positron emission tomography–computed tomography
FDG-uptake: Fluorodeoxyglucose
R-CHOP: Rituximab, cyclophosphamide, doxorubicin, vincristine, and prednisone
